# Salt tolerance in *Solanum pennellii*: antioxidant response and related QTL

**DOI:** 10.1186/1471-2229-10-58

**Published:** 2010-04-06

**Authors:** Anne Frary, Deniz Göl, Davut Keleş, Bilal Ökmen, Hasan Pınar, Hasan Ö Şığva, Ahmet Yemenicioğlu, Sami Doğanlar

**Affiliations:** 1 Department of Molecular Biology and Genetics, Izmir Institute of Technology, Urla 35430, Izmir, Turkey; 2 Alata Horticultural Research Institute, Erdemli 33740, Mersin, Turkey; 3 Biotechnology Program, Izmir Institute of Technology, Urla 35430, Izmir, Turkey; 4 Department of Food Engineering, Izmir Institute of Technology, Urla 35430, Izmir, Turkey

## Abstract

**Background:**

Excessive soil salinity is an important problem for agriculture, however, salt tolerance is a complex trait that is not easily bred into plants. Exposure of cultivated tomato to salt stress has been reported to result in increased antioxidant content and activity. Salt tolerance of the related wild species, *Solanum pennellii*, has also been associated with similar changes in antioxidants. In this work, *S. lycopersicum *M82, *S. pennellii *LA716 and a *S. pennellii *introgression line (IL) population were evaluated for growth and their levels of antioxidant activity (total water-soluble antioxidant activity), major antioxidant compounds (phenolic and flavonoid contents) and antioxidant enzyme activities (superoxide dismutase, catalase, ascorbate peroxidase and peroxidase) under both control and salt stress (150 mM NaCl) conditions. These data were then used to identify quantitative trait loci (QTL) responsible for controlling the antioxidant parameters under both stress and nonstress conditions.

**Results:**

Under control conditions, cultivated tomato had higher levels of all antioxidants (except superoxide dismutase) than *S. pennellii*. However, under salt stress, the wild species showed greater induction of all antioxidants except peroxidase. The ILs showed diverse responses to salinity and proved very useful for the identification of QTL. Thus, 125 loci for antioxidant content under control and salt conditions were detected. Eleven of the total antioxidant activity and phenolic content QTL matched loci identified in an independent study using the same population, thereby reinforcing the validity of the loci. In addition, the growth responses of the ILs were evaluated to identify lines with favorable growth and antioxidant profiles.

**Conclusions:**

Plants have a complex antioxidant response when placed under salt stress. Some loci control antioxidant content under all conditions while others are responsible for antioxidant content only under saline or nonsaline conditions. The localization of QTL for these traits and the identification of lines with specific antioxidant and growth responses may be useful for breeding potentially salt tolerant tomato cultivars having higher antioxidant levels under nonstress and salt stress conditions.

## Background

Soil salinity is a major environmental constraint to plant growth and productivity and is an especially serious problem in agricultural systems that rely heavily on irrigation [1,2]. A plant damaged by high salinity may suffer reduced shoot and root growth, yield losses and eventual death. These changes in plant growth are the result of salt's detrimental effects on plant physiology which include ion toxicity, osmotic stress, nutrient deficiency and oxidative stress [3]. Oxidative stress is, in fact, a secondary effect of salinity. Salt stress causes stomatal closure which reduces the carbon dioxide/oxygen ratio in plant cells. The excess oxygen in the plant is then used in the formation of reactive oxygen species (ROS) which, in turn, cause oxidative stress. Although reactive oxygen species such as the superoxide anion (O_2 _^.-^), hydrogen peroxide (H_2_O_2_), the hydroxyl radical (OH) and singlet oxygen (^1^O_2_) are produced and effectively neutralized during normal aerobic metabolism, ROS production increases to dangerous levels when a plant is under abiotic stress [3]. Excessive amounts of highly reactive ROS can damage proteins, lipids and nucleic acids by oxidation [4]. Therefore, it is critical that the plant counteract the production of reactive oxygen species with mechanisms for neutralizing them.

Antioxidant compounds (also called nonenzymatic antioxidants) such as phenolic compounds, ascorbic acid, tocopherols, glutathione and carotenoids are employed by plants to eliminate ROS. Phenolics are water-soluble antioxidants which readily neutralize ROS by donating their hydrogen atoms and are especially important because of their prevalence in plants and the significant contribution they make to water-soluble antioxidant activity [5]. There are more than 8,000 known phenolic compounds with flavonoids being the most common group of polyphenols in plants [6]. Although lipid-soluble antioxidants like carotenoids are also important scavengers of ROS, their relative contribution to total antioxidant activity in fruits and vegetables is much lower than the contribution from water-soluble antioxidants [6].

Antioxidant enzymes such as superoxide dismutase (SOD), catalase (CAT), ascorbate peroxidase (APX), and glutathione peroxidase (POX) scavenge ROS and are essential components of the plant's antioxidant defense system. Superoxide dismutase catalyzes the first step of the enzymatic defense mechanism, the conversion of superoxide anions to hydrogen peroxide and water. If superoxide anions are not neutralized, oxidation occurs and hydroxyl radicals are formed. Hydroxyl radicals are extremely harmful because they are very reactive and there is no mechanism for their systematic elimination. However, hydrogen peroxide can be decomposed by the activity of catalases and several classes of peroxidases which act as important antioxidants. As may be expected, expression of the genes for ROS scavenging enzymes is upregulated in plants under abiotic stress [7]. Moreover, the ability of certain species to increase production of antioxidant compounds and enzymes in response to salinity has been correlated with salt tolerance [8,9]. Various studies have also shown that genetically engineered plants containing higher levels of ROS scavenging enzymes, such as SOD [10], APX [11], and POX [12] have improved tolerance to abiotic stresses such as salinity.

Salt tolerance can be defined as the ability of plants to survive and maintain growth under saline conditions. Plants have three mechanisms to tolerate high salt concentrations: cellular homeostasis which includes ion homeostasis and osmotic adjustment; detoxification which includes neutralization of ROS; and growth regulation [13]. Knowledge of the genetic, physiological and biochemical control of these mechanisms is an essential step toward the development of crops with improved levels of tolerance to salt. Thus, the identification of genes, enzymes or compounds whose expression and/or production are altered by salt stress can perhaps aid in the breeding of salt tolerant cultivars [14-17]. Studies with barley, citrus, rice and tomato indicate that salt tolerance is a quantitative trait involving many genes and significant environmental effects [1].

Tomato is sensitive to moderate levels of salt stress and is produced in areas that are increasingly affected by salinity. Most of the wild relatives of tomato are easy to cross with cultivated tomato and provide a rich source of resistance and tolerance genes for biotic and abiotic stresses including salinity [18]. One of the objectives of this study was to determine the antioxidant responses of cultivated tomato, *Solanum lycopersicum *cv. M82, and the wild species, *S. pennellii*, upon exposure to salt stress. *S. pennellii *accession LA716 has been reported as salt tolerant in several studies [19-23]. The antioxidant response of these two tomato species to salt stress was assessed by measurement of antioxidant parameters including total water soluble antioxidant activity, total phenolic content, flavonoid content, and the activities of several antioxidant enzymes. Furthermore, a *S. pennellii *introgression line population was used to determine the vegetative growth response of plants to salt stress and to identify and map genes related to antioxidant accumulation under control conditions and in response to salt stress.

## Results

### Effects of salt on parental growth parameters

Typical of wild tomato species, *S. pennellii *accession LA716 grew more slowly than cultivated tomato under control conditions (see Additional file 1). Thus at the end of the experiment, LA716 plants were significantly shorter and, although they had more leaves, the wild species plants had much less leaf and root mass than *S. lycopersicum *cv. M82 plants. Exposure to salt stress resulted in statistically nonsignificant decreases in plant height and leaf dry weight in both *S. lycopersicum *and *S. pennellii*. Leaf number also decreased in both parents, however, only the change in the wild species was statistically significant. Stem diameter was also not significantly changed by salt treatment. The difference in root response to salt stress was quite dramatic in *S. lycopersicum *which suffered a 6.7-fold reduction in root dry weight while the wild species had a modest increase in root growth. However, the statistical significance of these differences could not be determined because replicate samples were bulked before drying. When the ratios between root and leaf dry weight were examined, it was seen that leaf growth was more sensitive to salt stress than root growth. For M82, the root to leaf mass ratio increased from 1.1 to 2.3, under salt stress, a 2.1-fold change. Similarly, for LA716 this ratio increased from 0.3 to 1.2, a 3.5-fold change.

### Effects of salt on parental antioxidant parameters

In nonstress conditions, *S. lycopersicum *had significantly higher levels than *S. pennellii *for six of the seven antioxidant parameters measured in this study (see Additional file 1). Total water-soluble antioxidant activity of M82, 681.1 μmol TE/100 g, was more than twice that of LA716, 307.6 μmol TE/100 g. Similarly, phenolic and flavonoid contents of cultivated tomato were 2.6 and 2.2-fold higher, respectively, than that of the wild species. Antioxidant enzyme activities were generally much higher in M82 than LA716. For example, APX activity of cultivated tomato under control conditions was 10.3-fold higher than that of the wild species. Similarly, CAT and POX activity were 8.4 and 6.5-fold higher, respectively, in M82 than LA716. SOD activity was the only exception as LA716 had 1.9-fold higher SOD activity than M82.

When grown in a saline environment, the wild species had significantly higher levels than M82 for all but three antioxidant traits: flavonoid content, CAT and APX activities (see Additional file 1). The greatest differences were observed in phenolic content, SOD and POX activities which were 1.6, 2.0 and 1.9-fold higher, respectively, in LA716 than M82.

When exposed to salt stress, M82 and LA716 had distinct antioxidant responses. In other words, each species experienced different changes in antioxidant levels due to salt stress. When subjected to salt stress, total water-soluble antioxidant activity and phenolic content decreased significantly for M82 (see Additional file 1). Whereas, flavonoid content increased slightly (1.3-fold) but significantly. For enzymatic antioxidants, salt stress resulted in insignificant increases in SOD and APX activity but more substantial decreases in CAT and POX activity (1.3 and 6.2-fold, respectively) in cultivated tomato. In comparison, the response of *S. pennellii *to salt stress was much simpler: all parameters increased significantly in LA716 when the plants were subjected to salinity. Thus, the average increase of total water-soluble antioxidant activity and antioxidants (phenolics and flavonoids) due to salt stress in the wild species was 2.4-fold. Even more dramatic amplifications in activity were observed in the enzymatic antioxidants of salt-stressed *S. pennellii*. Increases in activity ranged from 1.2 to 5.0-fold in the wild species; a far different response from that observed in the cultivar.

### Effects of salt on growth parameters of ILs

#### Plant height

Under control conditions, the ILs ranged in mean height from 14.3 cm (IL1-1) to 58.3 cm (IL2-4). Thirteen of the lines (25%) were shorter than M82 (32.3 cm) while the rest (75%) were taller than M82. Under salt conditions, the ILs ranged in mean height from 11.3 cm (IL1-1) to 50.7 cm (IL5-2). As with nonstress conditions, most of the lines (75%) were taller than M82 when grown in salt stress. In general, mean plant height was decreased by salt treatment. For the ILs, only one line (IL5-2) showed a significant increase in height (1.6-fold increase) when grown under salt conditions while 57% of the lines showed decreases and the rest showed no significant change. The largest decrease in height due to salt conditions, a 1.9-fold decrease, was seen in IL8-1.

#### Stem diameter

Under nonstress conditions, stem diameter of the ILs ranged from 4.0 mm (IL2-3, IL9-3) to 6.9 mm (IL10-3). Most of the lines (96%) had stem diameters that were smaller than that of M82 (6.7 mm). When grown under salt conditions, stem diameter of the ILs ranged from 3.3 to 7.0 mm for IL7-4-1 and IL8-1-1, respectively. Most of the lines (79%) had thicker stems than M82 (4.5 mm) under salt stress. However, very few significant changes in stem diameter were induced by salt treatment. Only 10% of the ILs showed significant decreases in stem diameter due to salt exposure and only 17% showed significant increases. The largest decrease in stem diameter was observed in IL 9-2 (a 1.8-fold decrease) and the largest increase was seen in IL8-1-1 (a 1.4-fold increase).

#### Leaf number

Average number of leaves on the ILs ranged from 6.3 (IL1-1, IL1-2) to 13.0 (IL2-1) under control conditions while M82 had an average of 9.0 leaves per plant. Thus, 50% of the ILs had more leaves than M82 and 50% had fewer leaves. When grown under salt stress, leaf number ranged from 5.3 (IL1-1) to 10.3 (IL2-1) for the ILs and was 7.3 for M82. Under salt stress, 64% of the ILs had more leaves than M82. Although leaf number decreased in most of the ILs under stress, only 2 ILs (5%) showed statistically significant decreases in this growth parameter. The largest reduction in number of leaves under salt conditions, 1.5-fold, was seen in IL1-3.

#### Leaf dry weight

Dry leaf weight of the ILs grown under normal conditions ranged from 0.26 (IL1-2, IL1-3) to 3.94 g (IL2-1). Most dry leaf weights of the ILs (73%) were lower than that of M82, 1.52 g. Under salt stress, leaf dry weight of the ILs ranged from 0.14 (IL1-3) to 3.30 g (IL2-1) while M82 had a dry weight of 1.10 g. Most ILs (83%) had dry leaf weights less than that of M82 under salt stress. In most of the ILs, dry leaf weight decreased under salt stress. IL10-2 had the greatest decrease, 9.5-fold, while IL 6-1 had the greatest increase, 2.4-fold. However, the significance of these differences could not be assessed because, as with roots, leaf samples from replicates were pooled before drying.

#### Root dry weight

Dry root weight for the ILs ranged from 0.10 (IL5-1) to 1.35 g (IL11-2) under nonstress conditions. M82 had the highest root dry weight, 1.68 g. Under salt stress, dry root weight ranged from 0.15 (IL3-5) to 1.85 g (IL11-1) for the ILs and 77% of the ILs had dry root weights greater than M82 (0.25 g) under stress conditions. The greatest decrease in the ILs was 3.3-fold in IL7-3. The greatest increase in root dry weight under stress was seen in IL5-1 which had a 3.5-fold increase. However, as stated previously, the significance of these differences could not be determined.

### Effects of salt on nonenzymatic antioxidants of ILs

#### Total water-soluble antioxidant activity

Antioxidant activity of the ILs under control conditions ranged from 293.0 (IL2-1) to 1407.7 (IL6-1) μmol TE/100 g. Most (71%) of the ILs had constitutive antioxidant capacities that were lower than that of M82. The ILs antioxidant activity when grown in salt ranged from 331.9 (IL2-6) to 996.4 (IL12-2) μmol TE/100 g. A total of 67% of the lines had antioxidant activity lower than M82 under salt stress. Antioxidant activity decreased significantly in 32% of the ILs and increased significantly in 46% of the lines under salt conditions. Eleven of the lines (22%) showed no significant change in antioxidant activity when grown in salt conditions. The greatest increase in antioxidant activity under salt stress was seen in IL2-1 (2.4-fold) while the greatest decrease was seen in IL6-1 (3.1-fold).

#### Total phenolic content

Phenolic content of the ILs ranged from 98.8 mg/kg (IL2-4) to 714.5 mg/kg (IL6-1) when grown under control conditions. Most of the lines (92%) had mean phenolic content lower than that of M82 which was 558.9 mg/kg. Only four lines (8%) had phenolic content higher than M82. When the lines were treated with salt, phenolic content ranged from 231.5 mg/kg (IL2-3) to 580.6 mg/kg (IL1-1) with 33 lines (66%) having higher phenolic content than M82 under salt conditions (330.6 mg/kg). Phenolic content of the ILs under salt stress decreased significantly in 60% of the lines and increased in 38% of the lines. The phenolic content of only one line (IL12-4) was not significantly affected by salt treatment. The greatest increase in phenolic content was measured in IL2-4 which had a 3.3-fold increase due to salt stress. The greatest decrease in phenolic content was observed in IL6-1 which had a 3-fold decrease in content.

#### Flavonoid content

Flavonoid content of the ILs ranged from 16.2 mg/kg in IL7-5 to 85.6 mg/kg in IL 6-1. The majority (74%) of lines had flavonoid content lower than that of M82. Flavonoid content of the ILs grown under salt stress ranged from 20.5 mg/kg (IL 4-4) to 95.9 mg/kg (IL11-1). Similar to control conditions, 76% of the ILs had flavonoid content lower than that of M82. Flavonoid content tended to increase under salt stress with 74% of the lines showing significant increases and 22% showing decreases. Only two lines (IL2-6 and IL12-4) were not significantly affected by salt stress. The greatest increase, 4-fold, was seen in IL5-4. The greatest reduction in flavonoid content due to salt treatment, 3.2-fold, was seen in IL6-1.

### Effects of salt on enzymatic antioxidants of ILs

#### Superoxide dismutase activity

SOD activity of the ILs ranged from 43.4 (IL5-4) to 52.3 (IL7-3) U/g leaf when grown under control conditions. Most (93%) of the ILs had higher SOD activities than M82, 44.7 U/g leaf. When treated with salt, SOD activity of the ILs ranged from 42.1 (IL4-3) to 57.1 (IL6-3) U/g leaf while M82 had an activity of 47.9 U/g leaf. Again, most of the ILs (90%) had higher SOD activities than M82 under salt conditions. A total of 57% of the lines showed increased activity, 11% showed decreased activity and 33% showed no significant change in SOD activity under salt stress. The greatest increase and decrease in SOD activity were only 1.2-fold, for IL5-4 and IL4-3, respectively.

#### Catalase activity

Under control conditions, catalase activity of the ILs ranged from 192,150 (IL6-1) to 1,470,936 (IL12-2) U/g leaf. Compared to M82, 81% of the ILs had lower CAT activity and 19% had higher activity. Under salt conditions, CAT activity of the ILs ranged from 191,688 (IL 4-3) to 782,256 (IL 2-2) U/g leaf while M82 activity was 605,880 U/g leaf. Compared to M82, 80% of the ILs had lower CAT activity and 20% of the lines had higher CAT activity. For the ILs, salt treatment significantly decreased activity in 71% of the lines, increased it in 23% of the lines and had no effect in the remaining 6% of the lines. IL7-1 had the greatest increase in CAT activity, 3-fold, while IL11-2 had the greatest decrease, 4.5-fold.

#### Ascorbate peroxidase activity

Ascorbate peroxidase activity of the ILs ranged from 97,566 (IL4-2) to 2,214,576 (IL2-2) U/g leaf. Most (96%) of the ILs had lower APX activities than M82 under control conditions. When grown in salt conditions, APX activities of the ILs ranged from 217,566 (IL11-4) to 2,372,568 (IL11-1) U/g leaf with 96% of the lines having activity lower than that of M82. Overall, 70% of the ILs showed a significant increase, 18% showed a decrease and 12% showed no change in APX activity under salt conditions. IL11-1 had the largest increase in activity, a 9.2-fold increase, while IL6-1 had the largest decrease, a 4.4-fold decrease.

#### Peroxidase activity

Peroxidase activity of the ILs under control conditions ranged from 167,334 (IL12-2) to 2,436,000 (IL6-1) U/g leaf. M82 had high POX activity as compared to the ILs, 2,102,760 U/g leaf. As a result, nearly all (98%) of the ILs had POX activity lower than that of M82. Under salt stress, POX activity of the ILs ranged from 151,200 (IL1-1) to 753,780 (IL12-1) U/g leaf. After salt treatment, 63% of the ILs had activities higher than that of M82. Significant decreases in POX activity were seen in 33% of the ILs. In contrast, increases in activity were observed in 59% of the ILs. No significant change in activity was seen in 8% of the lines. The greatest increase in POX activity, a 3.1-fold increase, was seen in IL8-1. The greatest decrease was seen in IL6-1, a 6.2-fold decrease. Interestingly, this is the same line that had the greatest decreases for phenolic, flavonoid and antioxidant contents as mentioned above.

### Correlations

Correlation analysis was performed to determine the relationship between the values obtained for each trait under control and salt conditions (Table 1). For the growth parameters, plant responses under stress and nonstress conditions were generally strongly correlated with the highest correlations observed for plant height (r = 0.80) and root dry weight (r = 0.72). The only exception was stem diameter which did not show a significant correlation between values for control and salt conditions. Interestingly, only one of the antioxidant parameters, total antioxidant capacity, was significantly correlated under stress and nonstress conditions (r = 0.48).

**Table 1:** Correlations (P < 0.05) between control and salt conditions for plant growth and antioxidant parameters.

**Parameter**^1^	Correlation
PLHT	0.80
STEM	ns
LNO	0.54
LDW	0.65
RDW	0.72
AOX	0.48
PHE	ns
FLA	ns
SOD	ns
CAT	ns
APX	ns
POX	ns

^1^Physiological trait abbreviations are: PLHT for plant height, STEM for stem diameter, LNO for leaf number, LDW for leaf dry weight, RDW for root dry weight.

Additional correlation analyses were done to examine the relationships between the different traits under both control and salt conditions (Tables 2 &3). For plant growth under nonstress conditions, the strongest correlation was observed between stem diameter and root dry weight (r = 0.54; Table 2). This correlation was much weaker under salt stress (r = 0.28; Table 3). In contrast, other growth traits showed stronger correlations under salt stress than in the control environment. Thus, root dry weight was not significantly correlated with leaf number or leaf dry weight under nonstress conditions; however, when plants were placed under salt stress, root dry weight became significantly correlated with these two traits (r = 0.46 and 0.30, respectively; Table 3).

**Table 2:** Correlations (P < 0.05) between growth and antioxidant parameters for plants grown under control conditions.

Parameter	STEM	LNO	LDW	RDW	AOX	PHE	FLA	SOD	CAT	APX	POX
**PLHT**	ns	ns	ns	ns	-0.43	ns	-0.45	ns	ns	-0.49	ns
**STEM**		ns	0.34	0.54	0.45	0.29	ns	ns	0.32	0.33	ns
**LNO**			0.44	ns	ns	ns	ns	ns	ns	ns	ns
**LDW**				ns	ns	ns	ns	ns	ns	ns	ns
**RDW**					0.54	0.37	0.32	ns	ns	ns	ns
**AOX**						0.66	0.73	ns	ns	0.53	0.47
**PHE**							0.46	ns	ns	0.47	0.35
**FLA**								ns	ns	ns	0.36
**SOD**									0.41	ns	ns
**CAT**										ns	-0.33
**APX**											0.33

**Table 3:** Correlations (P < 0.05) between growth and antioxidant parameters for plants grown under salt conditions.

Parameter	STEM	LNO	LDW	RDW	AOX	PHE	FLA	SOD	CAT	APX	POX
**PLHT**	0.43	ns	ns	ns	-0.38	-0.31	-0.40	ns	ns	ns	0.32
**STEM**		ns	ns	0.28	ns	ns	ns	ns	ns	-0.38	ns
**LNO**			0.61	0.46	ns	ns	ns	ns	ns	ns	ns
**LDW**				0.30	0.44	ns	ns	ns	ns	ns	ns
**RDW**					ns	0.30	ns	ns	ns	ns	-0.38
**AOX**						0.31	0.38	-0.35	ns	0.43	ns
**PHE**							0.67	ns	ns	0.33	-0.36
**FLA**								ns	ns	0.35	-0.34
**SOD**									ns	ns	ns
**CAT**										ns	ns
**APX**											ns

Under control conditions, there were strong positive correlations between antioxidant compounds (Table 2). The highest correlation (r = 0.73) was observed between total water-soluble antioxidant activity and flavonoid content while antioxidant activity and phenolic content were correlated at r = 0.66. Interestingly, under salt stress, although these correlations were still statistically significant, they were much weaker (r = 0.31 to 0.38; Table 3). These results may indicate that phenolic compounds with the highest antioxidant activity are consumed when plants are grown in saline conditions, thereby giving a different phenolic profile under salt stress. Flavonoid and phenolic contents were only moderately correlated under nonstress conditions (Table 2) but more strongly associated under salt stress (r = 0.67; Table 3). Antioxidant enzymes generally showed nonsignificant correlations among each other and moderate correlations with the other antioxidant compounds (Table 2 &3).

In general, strong correlations were not observed between growth and antioxidant parameters (Tables 2 &3). Interestingly, plant height had moderate statistically significant correlations with five of the seven antioxidant traits (AOX, FLA, and APX under control conditions; AOX, PHE, FLA and POX under salt stress) and all but one of these correlations (POX) was negative. Thus, taller plants tended to have lower antioxidant concentrations. Root dry weight had modest positive correlations with total water-soluble antioxidants, phenolics and flavonoids under control conditions (Table 2); however, these relationships weakened under stress (Table 3).

### Identification of QTL

#### QTL for total antioxidant activity

For total water-soluble antioxidant activity, 35 QTL were identified in the ILs (see Additional file 2, Figures 1, 2 &3). Among these, 11 QTL (31%) were detected in both salt and control conditions. For eight of these QTL (73%) *S. pennellii *alleles controlled decreased antioxidant activity. The average magnitude of effect of the wild alleles for these loci was approximately 50%. *S. pennellii *alleles for 13 (72%) of the 18 QTL detected under control conditions decreased the antioxidant activity of ILs. Whereas for the other five QTL, wild alleles were associated with increased antioxidant activity. The QTL *aox-c6.1 *had the highest magnitude of effect, a 107% increase in antioxidant activity under control conditions. Under salt conditions, six QTL associated with total antioxidant activity were detected. *S. pennellii *alleles for half of these QTL had total antioxidant activity at least 35% lower than M82, while for the other QTL, wild alleles specified higher activity ranging from 34 to 60%.

**Figure 1:**
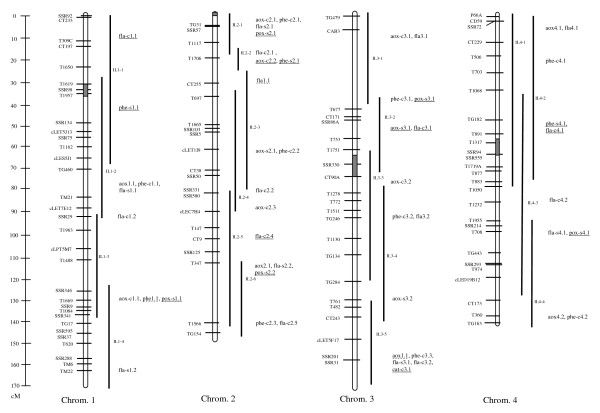
**Linkage map for chromosomes 1 to 4 of the IL population showing the locations of QTL identified in this work**. For loci that are underlined, *S. pennellii *alleles were associated with increased content/activity. Wild alleles for non-underlined loci were associated with decreased content/activity. Dotted underline indicates that the wild alleles were associated with both increased and decreased content/activity, depending on the environment (control or salt).

**Figure 2:**
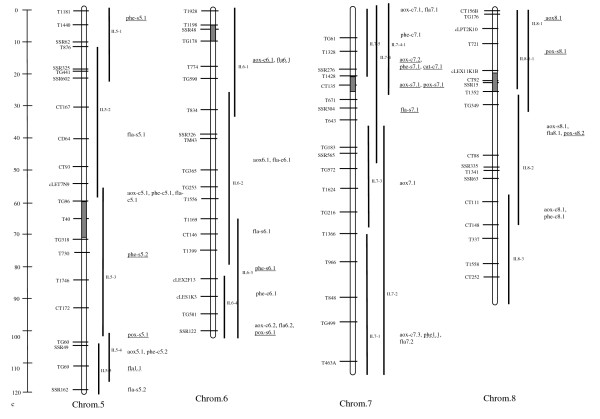
**Linkage map for chromosomes 5 to 8 of the IL population showing the locations of QTL identified in this work**. For loci that are underlined, *S. pennellii *alleles were associated with increased content/activity. Wild alleles for non-underlined loci were associated with decreased content/activity. Dotted underline indicates that the wild alleles were associated with both increased and decreased content/activity, depending on the environment (control or salt).

**Figure 3:**
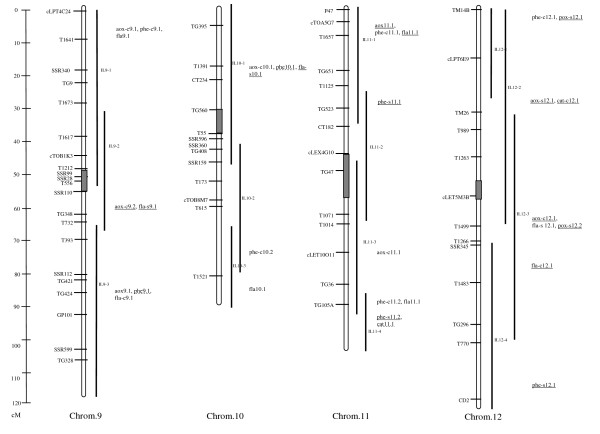
**Linkage map for chromosomes 9 to 12 of the IL population showing the locations of QTL identified in this work**. For loci that are underlined, *S. pennellii *alleles were associated with increased content/activity. Wild alleles for non-underlined loci were associated with decreased content/activity. Dotted underline indicates that the wild alleles were associated with both increased and decreased content/activity, depending on the environment (control or salt).

#### QTL for phenolic content

A total of 32 QTL were identified for phenolic content of the ILs (Figures 1, 2 &3). Of these QTL, 5 (16%) were effective under both control and salt conditions. The wild alleles for these loci had opposite effects on this trait under control and salt conditions such that the *S. pennelii *allele for each QTL was associated with decreased phenolic content in control conditions and increased content in stress conditions. Under salt stress, wild alleles for two of these loci, *phe9.1 *and *phe11.1*, were associated with increases in phenolic content of more than 70% as compared to M82. Under control conditions, 18 QTL were detected in the ILs which all had phenolic content at least 32% lower than M82. The greatest magnitudes of effect were observed for *phe-c2.2 *and *phe-c8.1*. For these loci, *S. pennellii *alleles were associated with 82 and 73% decreases in phenolic content, respectively. Nine salt-specific QTL were identified in the ILs. Wild alleles for all of these loci were associated with increases in phenolic content as high as 92% (*phe-s7.1*).

#### QTL for flavonoid content

Overall, 42 QTL were identified for flavonoid content (Figures 1, 2 &3). Among these QTL, 13 (31%) were detected under both control and salt conditions. *S. pennellii *alleles for the majority of these loci (69%) were responsible for decreased flavonoid content under both control and salt conditions. In general the wild alleles had similar magnitudes of effect under both conditions. However, for *fla6.1*, the *S. pennellii *allele controlled a 87% increase and a 56% decrease in flavonoids under nonstress and stress conditions, respectively. A total of 15 QTL were identified for flavonoid content under control conditions. For five of the QTL, wild alleles were associated with increased flavonoid content. Magnitudes of effect for these loci were moderate and ranged from 33 to 64%. *S. pennellii *alleles for the other ten QTL were responsible for decreases in flavonoid content of at least 33%. A total of 14 QTL were identified for flavonoid content under salt conditions. For the majority of the QTL (79%), wild alleles decreased flavonoid content under salt conditions. These loci also had moderate effects on the phenotype.

#### QTL for superoxide dismutase activity

None of the ILs had significantly higher (>30%) SOD activity than M82 under control or salt conditions. As a result, no QTL with *S. pennellii *alleles controlling increases in this trait were identified in this experiment.

#### QTL for catalase activity

A total of four QTL were associated with increased CAT activity from the wild allele (Figures 1, 2 &3). Of these QTL, only one *(cat11.1*) was detected under both control and salt conditions. For this locus, the *S. pennellii *allele was associated with a significant but moderate increase (44%) in CAT activity under control conditions and a moderate decrease (37%) in activity under salt stress. A total of three QTL were detected for increased constitutive catalase activity. The wild allele for the locus with the highest magnitude of effect, *cat-c12.1*, was associated with an 84% increase in CAT activity under nonstress conditions. No salt-specific QTL for which the *S. pennellii *allele increased enzyme activity were detected.

#### QTL for ascorbate peroxidase activity

None of the ILs had significantly higher APX activities than M82 under control or salt conditions. Therefore, no QTL with *S. pennellii *alleles controlling increases in this trait were identified in this experiment.

#### QTL for peroxidase activity

No loci were identified for which the wild alleles caused increases in POX activity under control or both control and salt conditions. Only salt-specific QTL were detected (Figures 1, 2 &3). Of these 12 loci, four had magnitudes of effect greater than 90%. *S. pennellii *alleles for the two most effective loci, *pox-s7.1 *and *pox-s 12.1*, resulted in increases in enzyme activity of 108 and 122%, respectively.

## Discussion

### Growth response to salt stress

The salt tolerance and sensitivity of LA716 and M82, respectively, were apparent in their different growth responses to salt stress. All of the growth parameters for M82 were negatively affected by salt treatment. In contrast, the tolerant *S. pennellii *accession had a more variable response to salinity. LA716 was able to maintain plant height, leaf number and root mass more effectively than M82 while, at the same time, reducing overall leaf mass and increasing stem diameter. Similar results were observed by Cano et al. [24] who saw greater reductions in leaf and root growth in cultivated tomato as compared to *S. pennellii*. Thus, the tolerance of this wild species may be explained by adaptation/alteration of several growth parameters.

Alternatively, salt tolerance may be the result of one or two parameters such as *S. pennellii*'s increased root to shoot ratio or its ability to maintain root growth during salinity, thus insuring adequate water uptake in soil with reduced osmotic potential. The difference in response of the root and shoot to salinity has been previously observed in tomato and many other species [15,25,26]. Under salt stress, reduction of the shoot is observed as delayed leaf emergence and expansion and decreased leaf size [26]. The mechanism of this increased sensitivity of the shoot to salt stress is not known, however, it is hypothesized that the plant's reduction in leaf growth is an adaptive response to save water in soils with reduced osmotic potential (i.e. dry and saline soils) [26]. *In vitro *studies with tomato shoot apices found that while *S. lycopersicum *shoot tips did not develop roots in the presence of NaCl, *S. pennellii *apices rooted easily at salt concentrations as high as 210 mM [24]. In this experiment, shoot growth was not as sensitive to salinity. Based on these findings, Cano et al. [24] suggest that root growth is the most indicative parameter for salt tolerance. Our results, however, suggest that both root and leaf mass are important factors in tolerance.

It was expected that the growth of the ILs would be more similar to M82 than LA716 because the ILs are genetically more similar to *S. lycopersicum *than *S. pennellii *(each line contains only a single well-defined introgression from the wild species). Indeed, growth parameter means for the ILs under stress and nonstress conditions were generally nearer to the M82 means. However, the individual ILs exhibited a broad range of variation for each growth trait. For example, some of the IL plants were much shorter than LA716 while others were nearly twice as tall as M82. Such individual lines with values outside of the parental extremes are manifestations of transgressive segregation due to new combinations of alleles in the progeny lines. As for M82 and LA716, most of the ILs suffered reduced plant height, leaf number and leaf dry weight in response to salt stress. Surprisingly, however, more of the ILs had increased stem diameter as a result of salt stress, a response that was similar to that of LA716. Moreover, a high proportion (42%) of the ILs had greater root growth under salinity than under control conditions, a response that was characteristic of *S. pennellii*. Thus, although the ILs were genotypically more similar to the cultivated parent, their phenotypic responses were less predictable and depended on the specific introgression carried by each plant.

### Antioxidant response to salt stress

In this experiment, *S. lycopersicum *cv. M82 and *S. pennellii *accession LA716 were shown to have different antioxidant profiles under control and salt stress conditions and different antioxidant responses to salt stress. The differences in total antioxidant and phenolic content between the two species were opposite to those reported by Rousseaux et al. [27] who studied the fruit antioxidant content of these species under normal growth conditions and found that *S. pennellii *had higher antioxidant activity and phenolic content. This significant difference in results may be attributed to the fact that Rousseaux et al. [27] measured fruit, not leaf, antioxidants and/or that they grew their plants in the field while we grew plants in a climate-controlled greenhouse. In the field, plants are expected to be subjected to higher levels of stress and a more variable environment, both of which may be responsible for higher antioxidant content in the wild species in previous work. Plants grown in the field also showed significant year-to-year variation and more inter-line variability than plants grown in the greenhouse [27].

For enzymatic antioxidants under control conditions, we found that only SOD activity was higher in *S. pennellii *leaves than cultivated tomato. In contrast, Shalata and Tal [20] found that activities of all tested enzymatic antioxidants were constitutively higher in *S. pennellii *leaves than in cultivated tomato. Similar results were reported for nonstress levels of antioxidant enzymes in *S. pennellii *roots [9,22] and root plastids [21].

Although it has been reported that irrigation of cultivated tomatoes with seawater may result in enhanced fruit antioxidant activity [28,29] a similar effect was not seen in M82 leaves in which only flavonoid content increased after salt treatment. This difference in results suggests the importance of tissue, cultivar (genotype) and salt concentration in determining antioxidant response to salinity. When treated with salt, LA716 had higher levels than M82 for all but three antioxidant traits: flavonoid content, CAT and APX activities. Our results agree with previous work in which salt stress was associated with higher levels of enzymatic antioxidant activities in *S. pennellii *than in *S. lycopersicum*. These findings were demonstrated for leaves [9,22], roots [9,20,22], root plastids [21], root mitochondria and peroxisomes [23]. In the same studies, M82 generally showed decreased enzyme activities under stress which agrees with our results for CAT and POX.

Based on the accumulated body of research, the salt tolerance of *S. pennellii*, as compared to cultivated tomato, is hypothesized to be the result of better protection from ROS [9,20-23,30]. This enhanced protection is attributed to higher constitutive levels of enzymatic antioxidants and greater induction of these enzymes following salt stress. Our results suggest a similar but slightly more complex explanation. In our work, *S. pennellii *did not have an inherently higher level of antioxidant enzymes and compounds than *S. lycopersicum*. However when grown under salt stress, the antioxidant system of *S. pennellii *was induced at a much higher level. As with the previous research, these results suggest that the salt tolerance of *S. pennellii *is associated with greater salt-induction of the antioxidant system in the wild species as compared with cultivated tomato. This increased expression leads to the accumulation of higher levels of antioxidant compounds in the wild species and, thus, greater protection from the damage caused by the increase in ROS that results from salt stress.

Because the ILs are genetically akin to M82, it was expected that they would also be more similar to M82 for the antioxidant parameters and, in general, have comparable responses to salt stress. Indeed, the mean values of the ILs for the antioxidant traits under both nonstress and stress conditions were more similar to M82 than LA716 for eight of the 14 measurements made (seven parameters measured under two treatment conditions). Only three of the measurements for the ILs were closer to *S. pennellii *values than to *S. lycopersicum *levels: control POX activity control, salt flavonoid content and salt CAT activity. In addition, the means for three measurements were intermediate between the two parental lines: control phenolic content, control APX and salt APX activities. The ILs also showed a tremendous range in antioxidant parameters. The greatest variation in nonenzymatic antioxidants was seen in the phenolic content of the ILs grown under control conditions, 7-fold variation. For enzymatic antioxidants, APX activity showed the greatest differences between lines with 22-fold variation under nonstress conditions. Less variation was apparent in salt-treated lines: phenolic content and APX activity had 2.5 and 10-fold variation in the ILs, respectively. The response of the ILs to salt stress had similarities with both *S. lycopersicum *and *S. pennellii*, depending on the parameter under consideration. Like LA716, the majority of the ILs showed increases in total antioxidant activity, flavonoid content and all enzymatic antioxidants when exposed to salt. However, like M82, the majority of the ILs had decreased phenolic content and CAT activity under salt stress. The variable antioxidant content and diverse responses of the ILs to salt stress are the result of transgressive segregation. The appearance of transgressive segregation in the population is important because it reinforces the validity of the ILs as a mapping population for the traits of interest and also indicates that improvement in antioxidant and salt tolerance traits should be possible by selection and breeding of such transgressive lines.

### Quantitative trait loci controlling antioxidant content and response to salt stress

**Figure 4:**
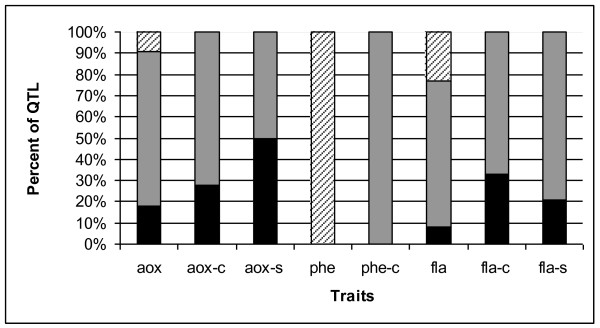
**Response of *S. pennellii *alleles for antioxidant trait QTL**. Proportion of loci for each antioxidant content trait with *S. pennellii *alleles showing an increase in both environments (black bars), decrease in both environments (gray bars) or opposite response in each environment (crosshatched bars).

A total of 125 QTL were identified for antioxidant content in this work. Thirty (24%) of these loci were responsible for antioxidant content when plants were grown under both control and salt conditions. The remainder, 54 (43%) and 42 (33%) loci, were detected only in control or salt-specific conditions, respectively. *S. pennellii *alleles for some of these loci had quite dramatic effects on nonstress and stress-induced antioxidant content. For example, the wild allele for *aox-c6.1 *was associated with a 107% increase in antioxidant activity as compared to the M82 allele while *phe-s7.1 *had a magnitude of effect of 92%. In general, the wild species alleles for the QTL showed the same direction of response to different environmental conditions. Thus, for the majority of loci that were detected under both control and salt conditions, *S. pennellii *alleles increased *or *decreased the trait under both conditions (Figure 4). An important exception is the phenolic content QTL. For these loci, *S. pennellii *alleles were always associated with decreased phenolics under control conditions and increased phenolics under salt stress (Figure 4). Other exceptions were 9% of the antioxidant activity loci and 23% of the flavonoid content loci.

Because part of the goal of this research was to identify alleles from *S. pennellii *that might be useful for breeding tomatoes with higher antioxidant content and perhaps better salt-stress tolerance, loci for which wild alleles were associated with increases in antioxidant compound content and enzymatic activity were of special interest. *S. pennellii *alleles for 18% of the QTL controlling total water-soluble antioxidant content under both control and salt stress environments were responsible for increased antioxidant activity (Figure 4). For loci identified only under control conditions, 28% of the QTL showed transgressive segregation with *S. pennellii *alleles responsible for increased antioxidant activity. Potentially useful wild alleles were also identified for total water-soluble antioxidant activity under salt stress with 50% of the loci showing positive effects from *S. pennellii*. All of the wild alleles for the phenolic content loci that were identified under both nonstress and stress conditions were responsible for increased phenolics under salinity. For flavonoid content, 33 and 21% of the QTL had wild alleles with transgressive segregation under control and salt conditions, respectively. Thus *S. pennellii *alleles for these loci were associated with increased flavonoid content (Figure 4). Alleles with such transgressive segregation should be useful for the development of higher antioxidant and salt tolerant tomatoes.

### Reliability of identified loci

QTL detection depends on many factors including but not limited to: population size, marker density of the linkage map, the accuracy and precision of phenotypic characterization, environment, and the method and threshold of detection. The *S. pennellii *ILs are an extremely useful tool for QTL analysis because they are a permanent population and provide whole-genome coverage with individual wild species introgressions in a cultivated tomato background. Thus, a locus can be pinpointed to a chromosomal location without concern for interaction with introgressions at other genomic sites. In the IL population, QTL detection is simplified and involves comparison of each line with M82, the background cultivar. In our study, an IL was considered to harbor a QTL if the trait mean for the line showed a 30% or greater difference in effect as compared to M82. Choice of a lower threshold would, of course, allow identification of more, less "effective" loci. However, 30% was chosen because it allowed the identification of major loci and is the threshold used by Rosseaux and coworkers in examining the genetic control of antioxidant traits in the *S. pennellii *ILs [27].

By comparison with this previous research, we can estimate the reliability of QTL detection in our work. Although Rosseaux and coworkers [27] identified loci involved in the antioxidant response of IL *fruit*, we found a remarkable degree of coincidence with our leaf antioxidant QTL. Four of the five loci identified by Rousseaux et al. were also detected in our work (*aox-c3.2*, *aox6.1*, *aox-c7.3*, and *aox-c10.1*). It is important to note that none of the matching antioxidant activity QTL were salt-specific; thus adding credibility to our findings because the loci were identified in similar, nonstress environments. Moreover, the most significant and consistently-detected (identified in all three years of the study) antioxidant activity locus identified in the previous work was found in IL6-2 on chromosome 6, the same location as our most significant antioxidant activity QTL, *aox-c6.1*. In both cases, the *S. pennellii *allele was responsible for highly significant increases in total water-soluble antioxidant activity. Rousseaux et al. [27] also examined phenolic content and identified nine loci, seven (78%) of which correspond to loci detected in our work:*phe-c3.1*, *phe-c3.2*, *phe-c5.2*, *phe-c7.1*, *phe7.1*, *phe-c8.1*, and *phe-c9.1*. Like the antioxidant activity QTL, these loci were identified in nonstress conditions. The correspondence between the QTL identified in this work and those described in previous work reinforces the credibility of selected loci and confirms the reliability of the QTL detection method used in both studies.

### Loci for breeding of higher antioxidant and potentially salt tolerant tomatoes

**Figure 5:**
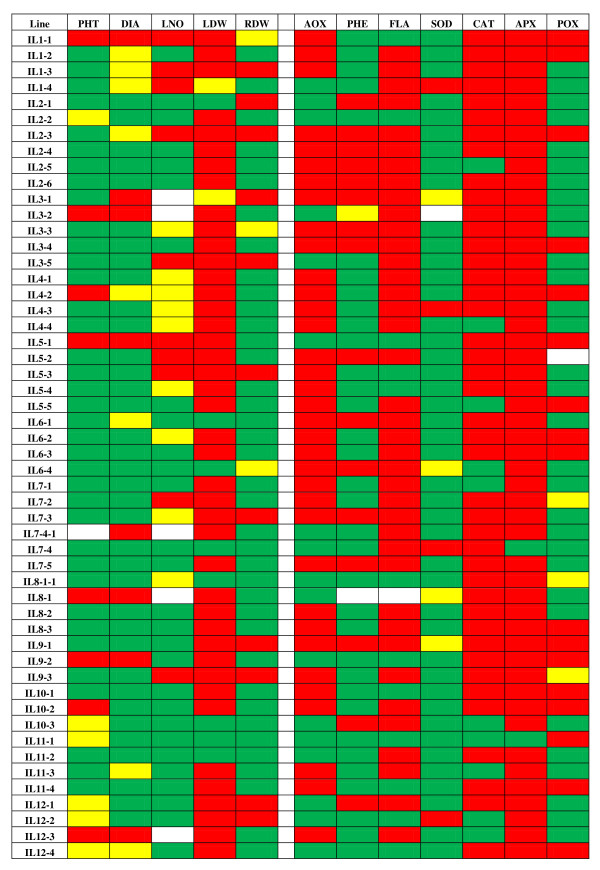
**Heat diagram showing the response of the ILs to salt stress as compared to M82**. Green boxes indicate traits for which the IL had a greater value than M82, red boxes indicate traits for which the IL had a lower value than M82, yellow boxes indicate traits with no appreciable difference and white boxes indicate missing data.

The antioxidant content loci identified in this work may be used for two purposes: to breed tomato cultivars that contain higher levels of antioxidants under normal growth conditions and to develop cultivars that produce higher levels of antioxidants in response to salinity and, thus, potentially have higher salt tolerance. The *S. pennellii *ILs are especially valuable for such breeding efforts. Because they are nearly isogenic with cultivated tomato and contain limited and genotypically well-defined introgressions, the ILs provide a convenient way to transfer identified traits to *S. lycopersicum *by backcross breeding and marker-assisted selection. In this work, ILs associated with increased antioxidant compound and enzyme activity under normal greenhouse conditions and salt stress were identified. Because both antioxidant content and salt tolerance are complex genetic processes, it is necessary to examine the potential salt tolerance of these ILs to determine which of the antioxidant loci are of most interest for improvement of salt tolerance in cultivated tomato. Figure 5 summarizes the growth and antioxidant responses of the ILs to salt stress as compared to M82. When interpreting these data, it must be remembered that salt tolerance is only partially determined by alterations in growth and the antioxidant defense system. Moreover, it is possible that tolerance may be achieved in more than one way for these different parameters. For example, when growth parameters are considered, some individuals may express tolerance as the ability to reduce shoot growth while maintaining root mass while others may exhibit tolerance as the ability to continue shoot growth despite salt stress. Similarly when the antioxidant system is considered, salt tolerance may be exhibited by individuals with high absolute values of antioxidant compounds under stress conditions and/or by plants which show the strongest response to salinity by increased production of all or only key antioxidants. Thus, different growth and antioxidant strategies may give the same result: salt tolerance.

Based on QTL analysis, IL7-4-1 was shown to be of special interest. This IL contained five loci for which wild alleles specified increases in antioxidant parameters under both stress and nonstress conditions (Figures 1, 2 &3). In this genomic region, *aox-c7.2 *and *aox-s7.1 *were responsible for 46 and 34% increases in antioxidant activity under control and salt conditions, respectively. In addition, the *S. pennellii *allele for *phe-s7.1 *was associated with a 92% increase in phenolic content under salt stress. IL7-4-1 also harbored QTL for increased antioxidant enzyme activity: *cat-c7.1 *and *pox-s7.1 *which were responsible for 34 and 108% increases in these traits under nonstress and stress conditions, respectively. In terms of growth response, IL7-4-1 produced more roots than M82 under salt stress while at the same time reducing leaf mass, representing one of the potential growth strategies for salt tolerance (Figure 5).

IL6-1 might also be useful for breeding higher antioxidant tomato cultivars as it contained antioxidant activity and flavonoids content QTL that were respectively associated with 107 and 87% increases in these traits under control conditions. Although similar antioxidant compound increases were not observed under salt stress for this line, IL6-1 performed very well under salt stress when compared to M82 (Figure 5). This line was taller than M82 under stress and produced more leaves and roots suggesting that it carries some degree of salt tolerance despite a seemingly unfavorable antioxidant response to salt stress.

IL8-1 or IL8-1-1 could be employed for improvement of both constitutive and salt-induced antioxidant content as they carried an antioxidant activity locus (*aox8.1*) that was responsible for 61 and 56% increases in this parameter under nonstress and stress conditions, respectively. This IL also contained a POX locus for increased peroxidase activity under salt stress. Interestingly, these two lines had quite different growth responses to salt stress. IL8-1, which has a larger introgression from *S. pennellii*, had reduced shoot growth compared to M82 while IL8-1-1 had greater shoot and root growth than the control. These results suggest that IL8-1 may be carrying alleles from *S. pennellii *that limit plant growth. Such linkage drag is very common in populations derived from wild species; however, the ILs are an excellent starting point for breaking this linkage as subILs can be easily generated by crossing of the line of interest with M82 or another cultivar of interest.

Figure 5 also shows that many more genomic regions of potential interest were identified in this work which should be useful for a greater understanding of the salt tolerance mechanism(s) of tomato. For example, under salt stress IL11-1 had greater content than M82 for all antioxidants except SOD (Figure 5). This line also had more root and shoot growth than M82 suggesting that it is salt tolerant. Several ILs conferring increased activity for the different antioxidant enzymes could be pyramided into the M82 background to assess the combined effect of these loci on salt tolerance, plant physiology and/or growth. In addition, individual lines showing a range of growth and/or antioxidant responses to salinity could be selected and their salt tolerance studied in more depth in order to obtain a better understanding of the different mechanisms of salt tolerance in tomato. Thus, the ILs or derived lines may be useful in pinpointing the exact strategy or strategies employed by tomato to achieve salt tolerance.

## Conclusions

In this research, loci related to antioxidant content and the response of tomato antioxidants to salt-stress were identified. Although these QTL may be useful for the development of higher antioxidant tomato cultivars, whether or not they will confer salt tolerance in the field is a separate consideration. As our results show, the antioxidant profiles, salt-induced antioxidant responses and growth responses of *S. lycopersicum*, *S. pennellii *and the ILs are complex. Although it was generally observed that salt stress resulted in higher levels of antioxidant compounds and enzymes in the wild species, a direct correlation between antioxidant levels and salinity tolerance is more difficult to prove. However, it is hoped that the results presented here have shed new light on the antioxidant responses of tomato and *S. pennellii *to salinity and that these results will be of interest to breeders as well as those studying the genetic and biochemical bases of salt tolerance.

## Methods

### Plant material, growth conditions and sampling

Fifty two *S. pennellii *tomato introgression lines (ILs) [31] and their parental lines, salt-sensitive cultivated tomato *S. lycopersicum *Mill. cv. M82 and the salt-tolerant wild species *S. pennellii *LA716, were used. Each IL contains a single introgression from *S. pennellii *in the genetic background of *S. lycopersicum *M82 such that the population provides complete coverage of the wild species genome [31].

Seeds were germinated in peat and plants were grown in aerated Hoagland's nutrient solution [32]. Six replicates of each plant line were grown. The experiment was carried out in a greenhouse with day/night temperatures of 27-29/23-25°C and relative humidity of 45-51%. Salt treatments were initiated when plants were at the seven true leaf stage and achieved with the gradual addition of NaCl to the nutrient solution. The first increment of salt was 25 mM and additional increments of 25 mM NaCl were added daily until the salt concentration reached the final treatment level of 150 mM NaCl. The plants were grown for 15 days at 150 mM NaCl. Thus, the total length of salt treatment was 21 days. After the treatment period, leaf samples were taken to be used in antioxidant assays. These samples were immediately frozen in liquid nitrogen and kept at -80°C until measurements were made. Plant height (cm), stem diameter (mm) and leaf number were determined for each plant. The remaining leaves and roots were harvested and combined for each line. After drying at 65°C for 48 hrs, leaf and root dry weights were determined (g).

### Nonenzymatic assays

To determine total water-soluble antioxidant activity (AOX), total phenolic (PHE) and flavonoid (FLA) contents, 1 g frozen leaf material was homogenized in 20 ml of cold distilled water using a Waring Blender fitted with an MC-1 stainless steel base. The homogenate was centrifuged at 16,000 g for 20 minutes at 4°C and the clear supernatant was used for measurements. For each extract, three replicate measurements were made. All assays were performed at 30°C using a Shimadzu spectrophotometer (Model 1700 UV Vis, Japan) equipped with a constant temperature cell holder.

Total water soluble antioxidant activity of tomato leaves was determined using the ABTS (2,2'-azinobis-3-ethyl-benzothiazoline-6-sulfonic acid) free radical-scavenging activity as described in Re et al. [33]. The ABTS radical cation stock solution was prepared by mixing 7 mM ABTS with 2.45 mM potassium persulfate followed by incubation in the dark for 12-16 hrs. Before use, this stock solution was diluted with phosphate-buffered saline (pH 7.4) to adjust its absorbance at 734 nm to 0.700 (± 0.02). For each sample, 2.5, 5.0 and 7.5 μl aliquots of tomato leaf extract were mixed with 2 ml ABTS radical cation solution and each reaction was kinetically monitored at 734 nm for 6 minutes with absorbances recorded at 1, 3 and 6 min. Three replicates were done for each aliquot volume. The free radical-scavenging activity was calculated as area under the curve (AUC). To calculate AUC, the percent inhibition/concentration values for the extracts and trolox standard were plotted separately against test periods (1, 3 and 6 min). The ratio of the area under the curves for each sample extract and the trolox standard curve (prepared using concentrations of 0.0075 to 0.045 μmol trolox per reaction) was determined and used to determine AUC. After these calculations, total water-soluble antioxidant activity was expressed as μmol TE (trolox equivalents)/100 g.

The Folin-Ciocalteu method of Singleton and Rossi [34] was used to measure total phenolic content. The method is based on the reducing power of phenolic hydroxyl groups with the Folin-Ciocalteu phenol reagent at 765 nm. Total phenolic content was expressed as gallic acid equivalents (mg gallic acid/kg fresh weight) based on a gallic acid standard curve. For flavonoid content determination, the method described by Zhishen et al. [35] was used. Absorbance was measured at 510 nm using an aluminum chloride colorimetric assay. Total flavonoids content was calculated based on an epicatechin standard curve (mg epicatechin/kg fresh weight).

### Enzymatic assays

To determine SOD, CAT (catalase), APX, and POX activities, 1 g frozen leaf material was homogenized in 20 ml of cold 100 mM sodium phosphate buffer (pH 6.8) and 0.1 g polyvinylpolypyrrolidone (PVPP) with a Waring Blender. The homogenate was centrifuged at 16,000 g for 20 minutes at 4°C. The clear supernatant was used for the measurement of enzyme activities with three replicates done for each assay. All enzyme assays were performed by spectrophotometric monitoring at 30°C using the spectrophotometer described above. SOD activity was assayed according to the method of Giannopilitis and Ries [36]. This assay monitors the ability of SOD to inhibit photochemical reduction of nitro blue tetrazolium (NBT) at 560 nm. One unit of SOD activity was defined as the amount of enzyme that caused 50% inhibition of nitro blue tetrazolium reduction per minute. The activities of CAT, APX and POX were assayed using the methods described by Lester et al. [37]. CAT activity was assayed by monitoring H_2_O_2 _decomposition at 240 nm. Monitoring of ascorbate oxidation at 290 nm was used to determine APX activity, while POX activity was determined by monitoring guaiacol oxidation at 470 nm. The activities of CAT, APX and POX were determined from the slope of the initial linear portions of absorbance vs. time curves. For these enzymes, the amount of enzyme causing a 0.001 change in absorbance per min was defined as one unit. The equations used for activity determinations were:

The final results of activity assays were expressed as units/g tissue.

### Statistical analyses and identification of QTL

Treatment means for the parental lines and ILs were compared with Student's t-test at P < 0.05. The effect of each introgression was determined by comparing its mean with the mean for M82 under both control and salt conditions with this value expressed as a percent (e.g., IL control mean/M82 control mean × 100). This value was then used to determine the difference in effect seen in the IL as compared to M82. The cultivar M82 is the genetic background for the ILs, thus, comparison with M82, allowed each difference in effect to be attributed to the particular introgression carried by the IL. For this calculation, M82 was set as 0% and 100% was subtracted from the percent obtained for each IL. Thus, a value of 50% in an IL would indicate that the introgression caused a 50% increase in the trait as compared to M82. For detection of QTL, a threshold of 30% was used. Thus, a QTL was assumed to be located in a particular introgression only if that introgression were associated with a 30% change in the trait as compared to M82. The use of a 30% threshold was chosen so that only QTL with large effects would be identified and to conform to the precedent of Rousseaux et al. (2005) who used this threshold to identify fruit antioxidant QTL in the *S. pennellii *ILs. For the antioxidant compounds, significant increases *and *decreases were used to identify loci controlling the parameters of interest. Thus, for these QTL, both positively (associated with an increase in antioxidant level) and negatively-acting (associated with a decrease in antioxidant level) *S. pennellii *alleles were detected. However, for the enzymatic antioxidants, only QTL with positively-acting wild species alleles are given in this work. Although negatively-acting QTL were detected, they are not given here because it is generally accepted that the salt tolerance of *S. pennellii *is associated with an increase in enzymatic antioxidants that is not seen in *S. lycopersicum*. Loci identified in only one of the treatments (control or salt) were assumed to be control- or salt-specific, respectively. QTL detected in both treatments were assumed to be important in controlling the trait of interest under both nonstress and stress conditions.

## Authors' contributions

AF analyzed the data and drafted the manuscript. DG assisted with data analysis and performed enzyme assays. DK and HP grew plants under stress and nonstress conditions and collected growth response data. BÖ and HŞ performed nonenzymatic antioxidant assays. AY assisted in the design of the study and coordinated antioxidant assays. SD conceived the study and participated in its design and coordination. All authors read and approved the final manuscript.

## Supplementary Material

Additional file 1 **Growth characteristics and antioxidant content of M82, LA716 and IL lines under control conditions and salt stress**. For M82 and LA716, salt effect refers to the fold change in trait/activity observed when lines were subject to salt stress as compared to control conditions. Salt effect values are only included for those differences which were statistically significant as determined by Student's t-test (P < 0.05). Nonsignificant effects are indicated by "ns", "na' indicates that statistical analysis was not appropriate because replicates were bulked. For the ILs, salt effect is the percentage of ILs showing significant increases and decreases in each parameter under salt stress as compared to nonstress conditions. Click here for file

Additional file 2 **Loci identified for the antioxidant traits in the IL population**. Control and salt-specific QTL names are suffixed with "c" and "s" respectively. Effect refers to the phenotypic effect (percent change relative to M82) of the *S. pennellii *allele for each locus. Click here for file
