# Supplementary material for: Salt tolerance in Solanum pennellii: antioxidant response and related QTL
Source: BMC Plant Biol. 2010 Apr 6;10:58. doi: 10.1186/1471-2229-10-58 (PMC2923532; doi:10.1186/1471-2229-10-58)
Supplement: Additional file 1 — Growth characteristics and antioxidant content of M82, LA716 and IL lines under control conditions and salt stress. For M82 and LA716, salt effect refers to the fold change in trait/activity observed when lines were subject to salt stress as compared to control conditions. Salt effect values are only included for those differences which were statistically significant as determined by Student's t-test (P < 0.05). Nonsignificant effects are indicated by "ns", "na' indicates that statistical analysis was not appropriate because replicates were bulked. For the ILs, salt effect is the percentage of ILs showing significant increases and decreases in each parameter under salt stress as compared to nonstress conditions. [file 1471-2229-10-58-S1.DOC]

**Additional file 1 - Growth characteristics and antioxidant content of M82, LA716 and IL lines under control conditions and salt stress.**

For M82 and LA716, salt effect refers to the fold change in trait/activity observed when lines were subject to salt stress as compared to control conditions. Salt effect values are only included for those differences which were statistically significant as determined by Student’s t-test (P<0.05). Nonsignificant effects are indicated by “ns”, “na’ indicates that statistical analysis was not appropriate because replicates were bulked. For the ILs, salt effect is the percentage of ILs showing significant increases and decreases in each parameter under salt stress as compared to nonstress conditions.

|  | **M82** | | | **LA716** | | | **ILs** | | |
| --- | --- | --- | --- | --- | --- | --- | --- | --- | --- |
| **Parameter** | **Control**  **Mean ± SE** | **Salt**  **Mean ± SE** | **Salt Effect** | **Control**  **Mean ± SE** | **Salt**  **Mean ± SE** | **Salt Effect** | **Control**  **Mean ± SE**  **(Range)** | **Salt**  **Mean ± SE**  **(Range)** | **Salt Effect** |
| **Plant Height (cm)** | 32.3 ± 3.7 | 23.7 ± 1.9 | ns | 22.5 ± 4.0 | 18.3 ± 1.7 | ns | 41.19 ± 1.57  (14.3-58.3) | 31.36 ± 1.26  (11.3-50.7) | 2% ↑  57%↓ |
| **Stem Diameter (mm)** | 6.7 ± 0.3 | 4.5 ± 0.3 | ns | 4.1 ± 0.6 | 3.1 ± 0.4 | ns | 5.03 ± 0.1  (4.0-6.9) | 5.08 ± 0.12  (3.3-7.0) | 17% ↑  10% ↓ |
| **Leaf Number** | 9.0 ± 0.6 | 7.3 ± 0.3 | ns | 11.0 ± 0.0 | 9.7 ± 0.3 | 1.1x ↓ | 8.8 ± 0.2  (6.3-13.0) | 7.7 ± 0.2  (5.3-10.3) | 0% ↑  5%↓ |
| **Leaf Dry Weight (g)** | 1.52 | 1.10 | na | 0.47 | 0.15 | na | 1.34 ± 0.1  (0.26-3.94) | 0.72 ± 0.08  (0.14-3.3) | na |
| **Root Dry Weight (g)** | 1.68 | 0.25 | na | 0.16 | 0.18 | na | 0.53 ± 0.04  (0.10-1.35) | 0.51 ± 0.05  (0.15-1.85) | na |
| **Total AOX Activity**  **(µmol TE/100g)** | 681.1 ± 5.8 | 620.9 ± 5.2 | 0.9x ↓ | 307.6 ± 4.1 | 670.4 ± 9.2 | 2.2x ↑ | 562.6 ± 33.8  (293.0-1,407.7) | 563.6 ± 25.9  (331.9-996.4) | 46% ↑  32% ↓ |
| **Total PHE Content (mg/kg)** | 558.9 ± 2.5 | 330.7 ± 1.4 | 0.6x ↓ | 216.7 ± 0.9 | 525.0 ± 1.1 | 2.4x ↑ | 356.5 ± 18.3  (98.8-714.5) | 393.25 ± 14.1  (231.5-580.6) | 38% ↑  60% ↓ |
| **FLA**  **Content**  **(mg/kg)** | 45.4 ± 0.2 | 60.0 ± 0.2 | 1.3x ↑ | 20.0 ± 0.1 | 53.0 ± 0.1 | 2.6x ↑ | 38.8 ± 2.5  (16.2-85.6) | 47.8 ± 2.8  (20.5-95.9) | 74% ↑  22% ↓ |
| **SOD**  **Activity**  **(U/g)** | 44.7 ± 0.6 | 47.9 ± 0.4 | ns | 84.1 ± 2.5 | 98.6 ± 2.1 | 1.2x ↑ | 48.0 ± 0.3  (43.4-52.3) | 50.2 ± 0.3  (42.1-57.1) | 57% ↑  11% ↓ |
| **CAT**  **Activity**  **(U/g)** | 798,984 ± 14,676 | 605,880 ± 6,858 | 0.8x ↓ | 95,190 ± 4,365 | 478,800 ± 29,097 | 5.0x ↑ | 650,172 ± 37,878  (192,150-1470936) | 445,536 ± 20,445  (191,688-782,256) | 23% ↑  71% ↓ |
| **APX Activity**  **(U/g)** | 1,718,280 ± 78,618 | 1965,600 ± 9621 | ns | 166,332 ± 14,028 | 279,300 ± 4,200 | 1.7x ↑ | 511,647 ± 63,777  (97,566-2,214,576) | 745,683 ± 57,291  (217,566-2,372,568) | 70% ↑  18% ↓ |
| **POX**  **Activity**  **(U/g)** | 2,102760 ± 59,391 | 339,150 ± 6,885 | 6.2x ↓ | 324,648 ± 206,784 | 644,700 ± 60,465 | 2.0x ↑ | 406,218 ± 51,114  (167,334-2436000) | 458,883 ± 57,417  (151,200-753,780) | 59% ↑  33% ↓ |
|  |  |  |  |  |  |  |  |  |  |
|  |  |  |  |  |  |  |  |  |  |
|  |  |  |  |  |  |  |  |  |  |
|  |  |  |  |  |  |  |  |  |  |
