# Supplementary material for: Salt tolerance in Solanum pennellii: antioxidant response and related QTL
Source: BMC Plant Biol. 2010 Apr 6;10:58. doi: 10.1186/1471-2229-10-58 (PMC2923532; doi:10.1186/1471-2229-10-58)
Supplement: Additional file 2 — Loci identified for the antioxidant traits in the IL population. Control and salt-specific QTL names are suffixed with "c" and "s" respectively. Effect refers to the phenotypic effect (percent change relative to M82) of the S. pennellii allele for each locus. [file 1471-2229-10-58-S2.DOC]

**Additional file 2- Loci identified for the antioxidant traits in the IL population.**

Control and salt-specific QTL names are suffixed with “c” and “s” respectively. Effect refers to the phenotypic effect (percent change relative to M82) of the *S. pennellii* allele for each locus.

| **Trait** | **QTL** | **IL** | **Effect (%)** |
| --- | --- | --- | --- |
|  |  |  |  |
| aox | *aox1.1* | 1-2 | -50(c), -43(s) |
|  | *aox2.1* | 2-5, 2-6 | -36(c), -47(s) |
|  | *aox3.1* | 3-5 | -43(c), 33(s) |
|  | *aox4.1* | 4-1 | -49(c), -44(s) |
|  | *aox4.2* | 4-4 | -47(c), -32(s) |
|  | *aox5.1* | 5-4, 5-5 | -39(c), -42(s) |
|  | *aox6.1* | 6-2 | -38(c), -44(s) |
|  | *aox7.1* | 7-3 | -40(c), -38(s) |
|  | *aox8.1* | 8-1, 8-1-1 | 61(c), 56(s) |
|  | *aox9.1* | 9-3 | -43(c), -44(s) |
|  | *aox11.1* | 11-1 | 37(c), 31(s) |
| aox-c | *aox-c1.1* | 1-3, 1-4 | -50 |
|  | *aox-c2.1* | 2-1 | -57 |
|  | *aox-c2.3* | 2-4, 2-5 | -50 |
|  | *aox-c2.2* | 2-2 | 41 |
|  | *aox-c3.1* | 3-1 | -40 |
|  | *aox-c3.2* | 3-3 | -43 |
|  | *aox-c5.1* | 5-2, 5-3 | -56 |
|  | *aox-c6.2* | 6-4 | -48 |
|  | *aox-c6.1* | 6-1 | 107 |
|  | *aox-c7.3* | 7-1, 7-2 | -52 |
|  | *aox-c7.1* | 7-5 | -39 |
|  | *aox-c7.2* | 7-4-1 | 46 |
|  | *aox-c8.1* | 8-2, 8-3 | -41 |
|  | *aox-c9.1* | 9-1 | -50 |
|  | *aox-c9.2* | 9-2 | 34 |
|  | *aox-c10.1* | 10-1 | -45 |
|  | *aox-c11.1* | 11-3 | -42 |
|  | *aox-c12.1* | 12-3 | 34 |
| aox-s | *aox-s2.1* | 2-3, 2-4 | -45 |
|  | *aox-s3.2* | 3-4 | -38 |
|  | *aox-s3.1* | 3-2 | 53 |
|  | *aox-s7.1* | 7-4, 7-4-1 | 34 |
|  | *aox-s8.1* | 8-2 | -35 |
|  | *aox-s12.1* | 12-2 | 60 |
|  |  |  |  |
| phe | *phe1.1* | 1-3, 1-4 | -55(c), 42(s) |
|  | *phe7.1* | 7-1, 7-2 | -66(c), 48(s) |
|  | *phe9.1* | 9-3 | -44(c), 73(s) |
|  | *phe10.1* | 10-1 | -62(c), 51(s) |
|  | *phe11.1* | 11-3, 11-4 | -37(c), 76(s) |
| phe-c | *phe-c1.1* | 1-2 | -55 |
|  | *phe-c2.1* | 2-1 | -58 |
|  | *phe-c2.2* | 2-3, 2-4 | -82 |
|  | *phe-c2.3* | 2-6 | -36 |
|  | *phe-c3.1* | 3-1, 3-2 | -50 |
|  | *phe-c3.2* | 3-3, 3-4 | -51 |
|  | *phe-c3.3* | 3-5 | -33 |
|  | *phe-c4.1* | 4-1, 4-2 | -53 |
|  | *phe-c4.2* | 4-4 | -73 |
|  | *phe-c5.1* | 5-2, 5-3 | -60 |
|  | *phe-c5.2* | 5-4, 5-5 | -65 |
|  | *phe-c6.1* | 6-3, 6-4 | -35 |
|  | *phe-c7.1* | 7-4, 7-5 | -57 |
|  | *phe-c8.1* | 8-2, 8-3 | -73 |
|  | *phe-c9.1* | 9-1 | -45 |
|  | *phe-c10.2* | 10-2, 10-3 | -51 |
|  | *phe-c11.1* | 11-1 | -35 |
|  | *phe-c12.1* | 12-1 | -36 |
| phe-s | *phe-s1.1* | 1-1, 1-2 | 76 |
|  | *phe-s2.1* | 2-2 | 70 |
|  | *phe-s4.1* | 4-2 | 44 |
|  | *phe-s5.1* | 5-1 | 40 |
|  | *phe-s5.2* | 5-3 | 42 |
|  | *phe-s6.1* | 6-3 | 35 |
|  | *phe-s7.1* | 7-4-1 | 92 |
|  | *phe-s11.1* | 11-1, 11-2 | 67 |
|  | *phe-s12.1* | 12-4 | 34 |
|  |  |  |  |
| fla | *fla2.1* | 2-3 | 69(c), -47(s) |
|  | *fla3.1* | 3-1 | -50(c), -56(s) |
|  | *fla3.2* | 3-3, 3-4 | -57(c), -55(s) |
|  | *fla4.1* | 4-1 | -37(c), -44(s) |
|  | *fla5.1* | 5-3, 5-4, 5-5 | -52(c), 46(s) |
|  | *fla6.1* | 6-1 | 87(c), -56(s) |
|  | *fla6.2* | 6-4 | -52(c), -45(s) |
|  | *fla7.2* | 7-1, 7-2 | -43(c), -32(s) |
|  | *fla7.1* | 7-5 | -64(c), -37(s) |
|  | *fla8.1* | 8-2 | -61(c), -38(s) |
|  | *fla9.1* | 9-1 | -53(c), -55(s) |
|  | *fla10.1* | 10-3 | -51(c), -46(s) |
|  | *fla11.1* | 11-1 | 39(c), 59(s) |
| fla-c | *fla-c1.2* | 1-2, 1-3 | -50 |
|  | *fla-c1.1* | 1-1 | 55 |
|  | *fla-c2.1* | 2-1, 2-2 | -45 |
|  | *fla-c2.2* | 2-4 | -34 |
|  | *fla-c2.5* | 2-6 | -43 |
|  | *fla-c2.4* | 2-5 | 37 |
|  | *fla-c3.2* | 3-5 | -44 |
|  | *fla-c3.1* | 3-2 | 64 |
|  | *fla-c4.2* | 4-3 | -33 |
|  | *fla-c4.1* | 4-2 | 39 |
|  | *fla-c5.1* | 5-2, 5-3 | -61 |
|  | *fla-c6.1* | 6-2 | -37 |
|  | *fla-c9.1* | 9-3 | -57 |
|  | *fla-c11.1* | 11-3, 11-4 | -45 |
|  | *fla-c12.1* | 12-3, 12-4 | 43 |
| fla-s | *fla-s1.1* | 1-2 | -46 |
|  | *fla-s1.2* | 1-4 | -35 |
|  | *fla-s2.1* | 2-1 | -38 |
|  | *fla-s2.2* | 2-5, 2-6 | -57 |
|  | *fla-s3.1* | 3-5 | -64 |
|  | *fla-s4.1* | 4-3, 4-4 | -66 |
|  | *fla-s5.1* | 5-2 | -36 |
|  | *fla-s5.2* | 5-5 | -55 |
|  | *fla-s6.1* | 6-2, 6-3 | -37 |
|  | *fla-s7.1* | 7-4 | 38 |
|  | *fla-s9.1* | 9-2 | 34 |
|  | *fla-s10.1* | 10-1 | 53 |
|  | *fla-s11.1* | 11-3 | -40 |
|  | *fla-s12.1* | 12-3 | -32 |
|  |  |  |  |
| cat | *cat11.1* | 11-4 | 44(c), -37(s) |
| cat-c | *cat-c3.1* | 3-5 | 30 |
|  | *cat-c7.1* | 7-4-1 | 34 |
|  | *cat-c12.1* | 12-2 | 84 |
|  |  |  |  |
| pox-s | *pox-s1.1* | 1-3, 1-4 | 96 |
|  | *pox-s2.1* | 2-1 | 78 |
|  | *pox-s2.2* | 2-5, 2-6 | 52 |
|  | *pox-s3.1* | 3-1, 3-2 | 92 |
|  | *pox-s4.1* | 4-3, 4-4 | 86 |
|  | *pox-s5.1* | 5-4 | 42 |
|  | *pox-s6.1* | 6-4 | 67 |
|  | *pox-s7.1* | 7-4, 7-4-1 | 108 |
|  | *pox-s8.1* | 8-1 | 74 |
|  | *pox-s8.2* | 8-2 | 70 |
|  | *pox-s12.1* | 12-1 | 122 |
|  | *pox-s12.2* | 12-3 | 44 |
